# Effect of External Use of Sesame Oil in the Prevention of Chemotherapy-Induced Phlebitis

**Published:** 2012

**Authors:** Nilufar Nekuzad, Tahereh Ashke Torab, Faraz Mojab, Hamid Alavi-Majd, Payam Azadeh, Gholamreza Ehtejab

**Affiliations:** a *Faculty of Nursing and Midwifery, International Branch of Shahid Beheshti University of Medical Sciences, Tehran, Iran .*; b *Army University of Medical Sciences, Tehran, Iran.*; c *Department of Nursing and Midwifery, Shahid Beheshti University of Medical Sciences, Tehran, Iran.*; d *Pharmaceutical Sciences Research Center, Shahid Beheshti University of Medical Sciences, Tehran, Iran. *; e *Department of Biostatistics, Faculty of Paramedicines, Shahid Beheshti University of Medical Sciences, Tehran, Iran.*; f *Department of Radiation Oncology, Imam Hossain Hospital, Shahid Beheshti Medical Sciences University, Tehran, Iran. *; g *Departmen of Radiation Oncology, Beasat Hospital, Armey Medical Sciences University, Tehran, Iran.*

**Keywords:** *Sesame Oil*, Prophylaxis, Phlebitis, Chemotherapy

## Abstract

Intravenous chemotherapy is an important mean for the treatment of cancers. Infusion phlebitis (Ph) is a common and acute complication of chemotherapy. The frequency of Ph is about 70% in patients undergoing chemotherapeutic management. It can induce the pain, increase the risk of thrombophlebitis, lead to incomplete follow-up, and thereby, affect the patient’s health status. Respecting the great importance of these issues, it is essential to prevent Ph.

This study conducted to determine the effect of external use of Sesame Oil (SO) in the prevention of Ph.

Sixty patients with colon or rectum cancer, who admitted for chemotherapeutic management, enrolled in clinical trial and were randomly divided into two equal groups: Control and Intervention. Ten drops of SO was applied twice a day for 14 days externally in intervention group, whereas the control group received nothing. Incidence and grade of Ph was measured in both groups. Data was analyzed through independent t-test, Χ2, Fisher’s exact test, Mann-Whitney, and Lagrange survival using SPSS 16.

The incidence of Ph was 10% and 80% in intervention group and control group, respectively.There was a significant difference between two groups (p < 0.05). Ph was 8 times more frequent in control group (R R = 8; AR R = 70%). In addition, there was statistically significant difference between the grade and incidence of Ph with SO and control group (p < 0.05).

According to these results, it seems that external use of SO is effective, safe and well-tolerated for prophylaxis from Ph. Therefore, it can be suggested as a selected prevention method for reducing the complication.

## Introduction

Chemotherapy is widely used as a systemic method in cancer treatment in which some “Cytotoxic” drugs are employed. These types of drugs have the aptitude of effective prohibition in the growth and development of cancerous cells (1), but the main problem which leads to the impediment of the cure is their adverse effects (2). Any chemotherapy lasting over 24 h, acts as an extremely powerful intravenous stimulant which usually causes “Phlebitis” which in turn leads to loss of superficial veins (3). Phlebitis is an inflammatory response to intravenously injected chemotherapy drugs that may last for weeks or months (4) and leads to various types of vein damage including, pain, erythema and swelling, warmth, hardening and thickening of injection area and finally, fever (5). The rate of chemotherapy-induced phlebitis incidence has been reported as 70% (6), which increases the probability of thrombophlebitis and embolism danger, affecting the health of patients (7), whereas, according to the standards of Intravenous Nurses Society, the accepted phlebitis amount for every society is 5% or less. Shielding and protecting the intravenous injection site is of great importance and vitality for nurses that should be able to detect and prevent the early symptoms of phlebitis as the first members of the healthcare team (8). Although presently there is no proved and precise method for preventing and treating chemotherapy-induced phlebitis (9), some preventative measures are proposed such as fast injection and diluting the chemotherapy, topical corticosteroid, or anti-inflammatory drugs, immediate catheter removal, applying warm wet compress on the site and then, redetecting the vein (10-13). Consequently, due to the importance of the matter, comprehensive studies over the application of a method of phlebitis prevention seems inevitable and plays a crucial role in changing the process of this complication.

One of the suggestions in this regard is using *Sesamum indicum*, the product of the medicinal and edible plant of sesame. Sesame has been long used in the traditional medicine of Iran and many other countries due to its antioxidant, anti-inflammatory and anti-bacterial significant effects (14, 15).

*S. indicum *has medicinal applications due to its resistance against the oxidation (16). It is also used in pharmaceutical industry as a useful solvent for some specific steroids and other solvable drugs in oil, capsules and oily injection products (17, 18).

In traditional medicine, sesame is used as a cure for asthma, hoarseness, Bowel obstruction, convulsion, eye disorders, itching, and burning with fire (19). It is anti-inflammatory and anti-rheumatism and also used as an antidote agent (17).

*S. indicum *also contains natural anti-oxidants which ruin the potential cancers in body and prevents gastrointestinal, prostate (the 2nd most common cancer among men) and breast (the 2nd most common cancer among women) cancers (20, 21). *S. indicum *also contains Vitamins E and F, crucial fatty-acids, which construct the skin layers, protecting the skin cells and defending the skin tissues from dehydration and destruction (19).

Regarding the therapeutic effects of *S. indicum*, national and international researches have been conducted, some of which have been studied here. One example is the research conducted by Hirsch *et al. *2008, in which the comparative effect of *S. indicum *herbal ointment and Flamazine for treating superficial burns was speculated. In this study, 40 patients in two groups were observed (one group “utilizing *S. indicum *herbal ointment” and the other “using Flamazine ointment”). The patients in each group used the related drugs on their burnt arms for 60 days. Then, the cases were checked regarding the pain relief, inflammation and repairing of the wheal improvement. The findings of this study manifested that from topical healing point of view, there were no meaningful difference between the two groups. It also revealed that using the *S. indicum *herbal ointment is more effective and can be a suitable replacement in curing the superficial burns, compared to the common medications (22).

Another survey was done with the purpose of specifying the effect of *S. indicum *and calcium hydroxide ointment on the non-surgical debridement time of third-degree burn wounds on a male rat and the result was that this sesame oil can decrease the healing time (14).

In the light of the results attained through the relevant researches performed, it is clear that the positive preventative effects of *Sesamum indicum *may result in a better tolerance of patients during the treatment process, leading to a reinforcement of the chemotherapy effect.

Seemingly, there has been no study conducted in Iran over the effect of this oil as a preventative measure against phlebitis, and for the same reason, this study was planned and performed.

## Experimental

The present study was performed as a randomized controlled clinical trial method on 60 patients under chemotherapy bedded in oncology section of Imam Husain Hospital (Tehran) in 2000.

The inclusion criteria for the patients in this study were as follows: consciousness, age range of 30-70, admitted to the oncology section of Imam Hussein Hospital, having colon or rectum cancer, being treated with Fluorouracil-5 only or together with other chemotherapy-related medications, not being diabetic, not having hypertension and autoimmunity, not having fever and neutropenia, not using antibiotic of any kind, not using analgesics and narcotics for the pain relief continuously, using no drugs or herbal oil to prevent phlebitis during the study, not applying any combination therapies such as radiotherapy during the study, not having any history of allergy to the sesame plant group, using the upper extremity for intravenously catheterization, applying No. 18-20 Angiocath of Supa Company for catheterization and following the required aseptic conditions in catheterization.

Samples, taking into consideration the 5% error probability of type 1 and the 0.2 difference of prevalence among the two groups and also considering the possible loss of samples in each group, were specified as 30. After acquiring the written permission from the Ethics Committee of International Branch of Shahid Beheshti University of Medical Sciences and Health Services and obtaining the written consent from the patients on which the research is performed, the samples were chosen randomly and were 30 for control group and 30 for intervention group.

The tools used for this study were as follows: a bipartite questionnaire with demographic information (age, sex, educational background) and some questions on the history of addiction, the duration of disease, the duration of chemotherapy, the chemotherapy diet (type, amount, time and the prescription type which was in the form of 4 protocols in this study), anti-inflammatory drug diet (which has been divided into two types regarding the type, amount and usage duration), and the absolute number of neutrophils; the checklist used for the measurement of phlebitis level based on the infusion therapy scale standards (2010) of the Royal Nursing College by which phlebitis is divided into six (0-5) distinct levels (23, 6, 24, 25). The later tool was used to check the phlebitis incidence and degree, before and after the intervention. To evaluate the questionnaire validity, the “content validity” and “face validity” methods were utilized. Simultaneous observation was also used to measure the constancy of the checklist; observation was done on 10 samples by two observers with similar characteristics and features using the same instructions which resulted in the correlation coefficient of 0.93.

The questionnaire was completed through the interview and file completion. The control group did not receive any *S. indicum*, whereas, the intervention group received 5 drops of it (100% pure *S. indicum*, manufactured by the Saman Sesame Oil Ltd. (Saman, Iran)) on the anterior forearm (around 10 cm toward the arm and in the path where the intravenous chemotherapy injections are done) every 12 h (morning and night before sleeping), from the 1st day of chemotherapy to the 14th day.

All the understudied cases were carefully speculated and followed up in these 14 days (during the hospitalization, after being released and at their next return to the hospital). Patients spotted with at least 2 signs of phlebitis (pain, erythema and swelling on the site), were referred to the phlebitis section for treatment. The data attained from the two groups were statistically compared and analyzed through descriptive statistics (including: median, mean, standard deviation and Kaplan-Meier estimation), inferential statistics (independent t-test, chi-square test, Fisher›s exact test, Mann-Whitney test, and Lagrange test) and SPSS 16 software.

## Results and Discussion

The distribution of understudied cases due to their demographic features and group-separated in Table 1, indicates that both groups have been identical regarding their sex, age, educational background, history of narcotic use, disease duration, chemotherapy period, chemotherapy diet type, anti-inflammatory drug diet type, and the absolute neutrophil count, and there has been no meaningful statistical difference between them (p < 0.05).

**Table 1:** Distribution of understudied cases due to their individual features and disease information, separated into two groups of Control and Intervention

**Group**			**Control (30)** **Intervention (30)**	
**Personal features**				
**Sex**	Female			
	Male	
Age (Years)*				
**Educational background**	Less than Diploma			
	Diploma or more	
**History of narcotic use**	Yes			
	No	
**Disease duration (Months)**				
**Chemotherapy period (Months)**				
Chemotherapy diet type**		Protocol (1)		
		Protocol (2)		
		Protocol (3)		
		Protocol (4)		
**Inflammatory drug diet type (Dexamethasone)**		Protocol (1)		
		Protocol (2)		
**Absolute neutrophil count**				

*Standard mean ± deviation; **All the chemotherapy protocols include 5-fluorouracil as the base drug, prescribed either alone or together with other drugs

**Figure 1:**
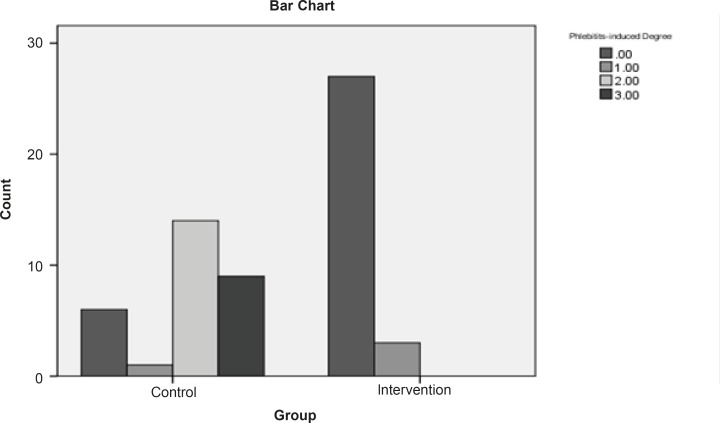
Frequency of phlebitis incidence in control and intervention groups

The findings manifested that 10% of the intervention group and 80% of the control group got afflicted with phlebitis (Figure 1). 

**Figure 2:**
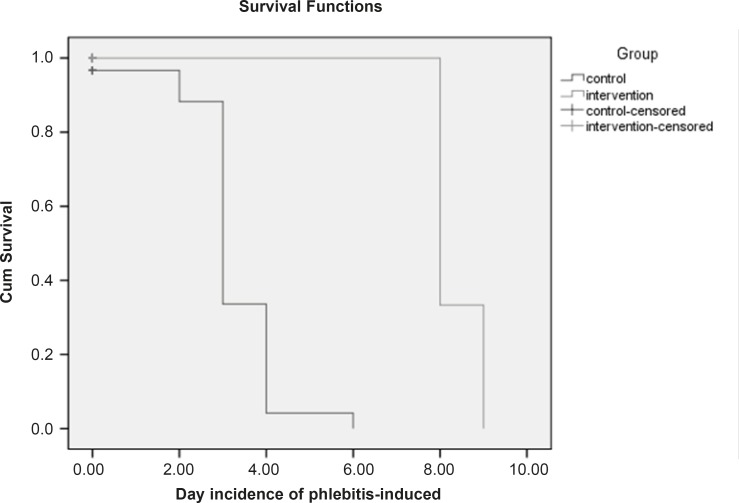
Kaplan-Meier survival curve on the two hospitalized and censored groups. (Censored group are those who stayed till the end of the study and were not afflicted with phlebitis). The survival time for the censored group was equal to the total days of the study (From the beginning till the end).

Based on the chi-square test, there is a meaningful difference between the two groups regarding the phlebitis incidence (p < 0.05). In control group, in which the *S. indicum *was not used, the relative risk of phlebitis incidence was 8 times more than the intervention group. In addition, the absolute risk reduction indicates that the phlebitis incidence in control group is 70% more in comparison with the intervention group. The mean of phlebitis incidence is 0.1 in intervention group and 1.9 in control group. Furthermore, Mann-Whitney test shows that there is a meaningful statistical difference (p < 0.05) between the mean of phlebitis incidence in the mentioned groups. Figure 2 shows that the survival time in control group is 80% till the 6th day. The non-parametric Lagrange test manifested that there is a meaningful difference between the two groups regarding the time of phlebitis incidence (p < 0.05). In other words, the time of phlebitis incidence in intervention group has been delayed.

## Conclusion

The present study showed that the use of Sesamum indicum in patients under chemotherapy has decreased the phlebitis incidence.

In various studies, the anti-oxidant, anti-mutagen and anti-inflammatory features of *S. indicum *have been reported (19, 25). In studies about the improvement of cough in children in range of 2-12 (years), it was revealed that in 95% of the children, using *S. indicum *has had positive effect on their coughs severity and frequency (26).

A survey over the so-far applied studies shows that no study has been performed on human cases, regarding the effect of *S. indicum *on chemotherapy-induced phlebitis. However, a comparison has been performed on the effect of Aloe Vera on chemotherapy-induced phlebitis. In the study done by Dong *et al. *(2001) to compare the effect of Aloe Vera with that of Magnesium Sulfate in preventing phlebitis in patients under chemotherapy, it was revealed that the phlebitis incidence in groups treated with Aloe Vera and Magnesium Sulfate was 7.3% and 25%, respectively. In other words, there has been a meaningful difference in the incidence of chemotherapy-induced phlebitis between the two groups (p < 0.01) (patients who had used Aloe Vera were affected with phlebitis less than those who had used Magnesium Sulfate) (28).

In the study conducted by Dai *et al. *(2007) on 259 patients in order to survey the effect of herbal poultice of Aloe Vera with Novocain (2%) injection to prevent chemotherapy-induced phlebitis on 259 patients under the treatment of Fluorouracil, 11.98% of the intervention group and 42.39% of control group got afflicted with phlebitis which showed a meaningful difference (p < 0.01) (29). The findings of the present study about the effect of using *S. indicum *in preventing the chemotherapy-induced phlebitis showed that the rate of phlebitis incidence using this oil is similar to that of the two mentioned studies. Considering the excess of this oil and the low price of it and also taking into consideration that it has been welcomed by the patients, this can be a very good solution for the patients in cancerous conditions. The present study also shows that the frequency of phlebitis incidence in intervention group is less than that of control group (there is a meaningful difference between them).

It should be noted that factors such as age, sex, type and the amount of chemotherapy are effective on the phlebitis incidence and its frequency (23, 6) (in this study, all these variables were statistically the same across the groups). Moreover, the meaningful statistical difference between the survival times of the two groups indicates that the applied intervention in the current study has delayed the phlebitis incidence in the intervention group. In conclusion, the findings show that the rate of phlebitis incidence in those who had not used the *S. indicum *was 70% more than that of those who used it. In this study, contrary to the teaching cases about protecting the skin, it was assumed that patients might not been careful about the health advice sanitary notes. The present study proves that using *S. indicum *can play an important role in preventing and delaying the chemotherapy-induced phlebitis incidence. Since chemotherapy is normally used in metastatic diseases where operation and radiotherapy have failed, preventing the adverse effects of it, can lead to a better tolerance from patients and efficacy of the treatment. Therefore, as the preventative and treatment role of *S. indicum *has been proved in the previous studies in skin problems and burns as an anti-inflammatory agent over the animals, coupled with the present findings, it seems that this oil has an important role in preventing and decreasing the phlebitis incidence, specifically as it is found abundantly in Iran and compared to the chemical drugs, has no adverse effects and is quite cheap.

A main reason for the conduction of this survey was to highlight the priority of shielding and protecting the under chemotherapy veins in adverse patients and its results can improve the situation at clinical, educational and research levels. It is suggested to work on the “effect of the external use of *S. indicum *on improving or decreasing the adversity of the phlebitis pain for the next researches. The finding of the present study can also be used as a step towards other researches in educational and research centers of nursing and can enlighten the important role of nurses in the preventive affairs.
